# Regulation of Melanopsins and *Per1* by **α**-MSH and Melatonin in Photosensitive *Xenopus laevis* Melanophores

**DOI:** 10.1155/2014/654710

**Published:** 2014-05-13

**Authors:** Maria Nathália de Carvalho Magalhães Moraes, Luciane Rogéria dos Santos, Nathana Mezzalira, Maristela Oliveira Poletini, Ana Maria de Lauro Castrucci

**Affiliations:** ^1^Department of Physiology, Institute of Biosciences, University of São Paulo, R. do Matão, Travessera 14, No. 101, 05508-900 São Paulo, SP, Brazil; ^2^Department of Physiology, Institute of Biological Sciences, Federal University of Minas Gerais, 31270-901 Belo Horizonte, MG, Brazil

## Abstract

**α**-MSH and light exert a dispersing effect on pigment granules of *Xenopus laevis* melanophores; however, the intracellular signaling pathways are different. Melatonin, a hormone that functions as an internal signal of darkness for the organism, has opposite effects, aggregating the melanin granules. Because light functions as an important synchronizing signal for circadian rhythms, we further investigated the effects of both hormones on genes related to the circadian system, namely, *Per1* (one of the clock genes) and the melanopsins, *Opn4x* and *Opn4m* (photopigments). *Per1* showed temporal oscillations, regardless of the presence of melatonin or **α**-MSH, which slightly inhibited its expression. Melatonin effects on melanopsins depend on the time of application: if applied in the photophase it dramatically decreased *Opn4x* and *Opn4m* expressions, and abolished their temporal oscillations, opposite to **α**-MSH, which increased the melanopsins' expressions. Our results demonstrate that unlike what has been reported for other peripheral clocks and cultured cells, medium changes or hormones do not play a major role in synchronizing the *Xenopus* melanophore population. This difference is probably due to the fact that *X. laevis* melanophores possess functional photopigments (melanopsins) that enable these cells to primarily respond to light, which triggers melanin dispersion and modulates gene expression.

## 1. Introduction

Color change is an important strategy for animals' camouflages, which allows them to match their body color with the environment [1]. In ectothermic vertebrates, pigment granule aggregation or dispersion, within the cytoplasm of cutaneous pigment cells, are responsible for the physiological color changes resulting in skin lightening or darkening, respectively [2, 3]. Notably, pigment translocation can be induced by light [4–7] and by several neurohumoral signals [7, 8].

*α*-Melanocyte stimulating hormone (*α*-MSH), which is produced in the pars intermedia of the pituitary gland, is a darkening agent in all vertebrates. It induces a very rapid dispersion in dermal melanophores of ectothermic vertebrates [9–13], and it also plays a role in skin, fur, and feather darkening [14, 15], in cell proliferation, in cell differentiation [16], and immune activity [17, 18] of endothermic animals. Human *α*-MSH is produced in sites other than the pituitary, such as the skin itself and its systemic action promotes melanin production by the human skin in response to UV light [19]. In addition, *α*-MSH is produced in the mammalian hypothalamus affecting behaviors such as food intake and lordosis [20, 21].

Melatonin (5-methoxy-N-acetyl tryptamine) is an indoleamine synthesized by the pineal gland and released at night. Contrary to *α*-MSH, melatonin aggregates melanin granules acting as a lightening hormone [7, 22]. Many ectothermic vertebrates exhibit a circadian rhythm of color change, a night paling related with melatonin secretion [23, 24]. Melatonin integrates the endocrine system and the external environment, translating the photoperiod information into hormone signal, thus synchronizing various aspects of physiology and neuroendocrine functions with light-dark cycles [25]. This hormone plays a crucial role in the regulation of circadian and seasonal changes, influencing reproductive functions through the hypothalamic-pituitary-gonadal (HPG) axis [26]. In addition, melatonin may also act as an antioxidant agent [27], some of its antioxidative activities being exerted by its metabolite, cyclic 3-hydroxymelatonin, which completely prevents the cytochrome C degradation by hydrogen peroxide [28].

Studies on the photoresponses of* Xenopus laevis* melanophores led to the discovery of the photopigment melanopsin, which is present in the retina of all vertebrates, including humans [29, 30]. In mammals, melanopsin is present in a subpopulation of photosensitive ganglion cells [31], which convey light information to the hypothalamic suprachiasmatic nuclei (SCN) [32, 33], the master biological clock. In addition to adjusting the biological clock to light-dark cycles, these cells mediate nonvisual photic responses, such as pupillary constriction and melatonin suppression [34, 35]. In 2006, Bellingham and coworkers [36] demonstrated the existence of two* Opn4* genes, encoding melanopsins in nonmammalian vertebrates. The second melanopsin,* Opn4m*, presents a much higher homology with the mammalian than with the* X. laevis* melanopsin, which was then named* Opn4x*. Despite the presence of melanopsins in nonmammalian retinas, their function as a photoentrainment pigment has yet to be defined.

The molecular basis for endogenous biological rhythm is a loop of transcriptional/translation of clock proteins. In general, CLOCK and BMAL1 dimers acting through E-box elements increase the transcription of* Per*,* Cry*,* Reverb-*α*,* and* Ror* which after being translated inhibit CLOCK/BMAL1 effect [37]. Additionally, CLOCK/BMAL1 dimers regulate other genes known as clock controlled genes [38–40]. Tyrosinase, a rate-limiting enzyme for melanin production, has E-box motif in its promoter, suggesting that it can be a clock-controlled gene [41].

Although *α*-MSH and melatonin have opposite effects on pigment translocation, both mediate light responses, that is, *α*-MSH disperses melanin granules in the same manner as light does, whereas melatonin is the messenger of the darkness. *α*-MSH and light have the same effect on pigment translocation within* X. laevis* melanophores; however, the intracellular signaling pathways are different [42, 43], which may indicate plasticity in this response. *α*-MSH acts through MC1R receptor coupled to a Gs-protein, triggering the activation of the cAMP pathway [44], whereas light stimulates melanopsin leading to phospholipid hydrolysis and increase of cytosolic calcium [42]. On the other hand, melatonin triggers* X. laevis* melanin aggregation through the activation of Mel 1c receptor, which is coupled to a Gi-protein, thus leading to a decrease in cAMP [22].

*X. laevis *melanophores incorporate all components of a circadian system, as they express clock genes and photopigments. In addition,* Per1* expression increases in response to a blue light stimulus, indicating the mediation of a blue sensitive pigment [45]. The fact that both hormones, *α*-MSH and melatonin, signal external environmental light conditions to the organisms in an opposite manner, and that light is an important zeitgeber in* X. laevis* melanophores, we hypothesized that these hormones may differentially affect the melanopsins* Opn4x* and* Opn4m* (blue sensitive photo-pigments) as well as* Per1* expression.

## 2. Material and Methods

Immortalized cultures of embryonic* X. laevis* melanophores (a kind gift of Professor Mark Rollag, University of New Mexico, USA) were kept in 60% L-15 medium, supplemented with 5 mg/mL insulin/transferrin/selenium, 2X hypoxanthine/thymidine, 1% antibiotic/fungicide solution (10,000 U/mL penicillin/10,000 *μ*g/mL streptomycin/25 *μ*g/mL amphotericin B), and 10% non-inactivated fetal calf serum (all from Life Technologies, Carlsbad, CA, USA) at pH 7.5 and 25°C [46]. The culture medium was changed weekly and when the cells reached 80% confluence, they were removed with 60% Tyrode/EDTA solution and transferred to new flasks (the cells from one flask were split into 3 flasks).

For the experiments, the cells were plated 2 × 10^6^ per 25 cm^2^ flask, the serum was reduced to 2%, and 2 × 10^−7^ M retinaldehyde was added. Cells were manipulated in the dark using Konex illuminator with a 7 W red light (~5 lux) and a Safe-Light GBX-2 filter (Kodak, SP, Brazil). Each experiment was repeated 2 to 3 times with duplicate flasks of cells (*n* number presented in the graphs).

The protocols described below were based on the fact that* Xenopus *melanophores aggregate the melanin granules in DD, or in response to melatonin, and disperses pigment granules in response to light or to *α*-MSH. Therefore, *α*-MSH was employed to mimic light signal in aggregated cells, that is, in DD, whereas melatonin was applied on already dispersed cells; there is LD.

### 2.1. Protocol 1: *α*-MSH Treatment

Cells were kept under constant darkness for 5 days. At 8:00 h of the fifth day, the flasks were divided into two groups: (1) cells were subject to a medium change without hormone (control); (2) cells were subject to a medium change containing 10^−9^ M *α*-MSH (Sigma-Aldrich, St. Louis, MO, USA). Twelve hours later, both groups received a new medium change without hormone. At 8:00 h of the sixth day (24 hours after the beginning of the treatment) total RNA was extracted every 4 hours (0, 4, 8, 12, 16, and 20 ZT).

### 2.2. Protocol 2: Melatonin Treatment

Cells were kept in 12 : 12 LD for 5 days and then treated for 6 hours with 10^−9^ M melatonin (Sigma-Aldrich, St. Louis, MO, USA) in one of the following conditions: (a) beginning of the light phase of the 6th day and (b) beginning of the dark phase of the 6th day. Control cells were kept in the absence of the hormone but were subject to similar light conditions and medium changes. Twenty-four hours after beginning of the treatment, total RNA was extracted every 4 hours (0, 4, 8, 12, 16, and 20 ZT). During the light phase, the flasks received irradiance of 87.85 µw/cm^2^ (600 lux, 2.5 × 10^14^ photons·s^−1^·cm^−2^, full spectrum, 8-W cool white fluorescent tube, T5-8W, SCT, São Paulo, Brazil).

### 2.3. RNA Extraction, RT-PCR and Quantitative PCR

Total RNA was extracted with TRIzol reagent; the RNA pellet was then resuspended in DEPC water and treated with DNase (*turbo-DNA-free*), according to the manufacturer's instructions (all reagents from Life Technologies, Carlsbad, CA, USA). RNA concentration was determined with a spectrophotometer (Nanodrop, Willmington, DE, USA) and 1 *μ*g was used in reverse transcription reaction with SuperScript III and random primers (Life Technologies, Carlsbad, CA, USA).

Quantitative PCR reactions were carried out with primers and probes specific for the genes of interest or ribosomal 18S, designed with* Primer Express *(Life Technologies, CA, USA) based on Genbank sequences (http://www.ncbi.nlm.nih.gov/PubMed/) and synthesized by IDT (Coralville, IA, USA) (Table 1). Multiplex quantification was performed for* Opn4x *and 18S RNA and* Per1* and 18S RNA; the solutions contained 1 µL of cDNA, primers (300 nM for each gene,50 nM for 18S RNA), probes (200 nM for each gene, 50 nM for 18S RNA), Platinum Supermix (Life Technologies, Carlsbad, CA, USA) supplemented to final concentrations of 6 mM MgCl_2_, 0.4 mM dNTPS, 0.1 U/µL Platinum Taq DNA polymerase.

To normalize the results, 18S ribosomal RNA was used, as recommended for mammalian and nonmammalian species [47–51]. The efficiency of each pair of primers was calculated and varied between 80 and 104%. The assays were performed in i5 thermocycler (BioRad Laboratories, Hercules, CA, USA), with the following conditions: 7 min at 95°C, followed by 40 cycles of 30 s at 95°C and 30 s at 55°C.

### 2.4. Statistical Analyses

The results were analyzed using the ΔΔCT method [52]. The threshold was established by the thermocycler software and crossed the amplification curves to determine the number of cycles, the CT. The difference between the CTs for 18S RNA and the CT for each gene at the same time point is the ΔCT. The maximal ΔCT was then subtracted from each ΔCT value to obtain ΔΔCT, used as a negative exponential of base 2 (2^−ΔΔCT^). The log values (at least four flasks of cells, from two independent experiments) were averaged and graphed as mean ± SEM relative to the minimal value expression for each protocol. The levels of significance of differences among time points were determined comparing the log data by one-way ANOVA, followed by Tukey; between time points in different protocols, as well as control versus hormone-stimulated groups, the log data were compared by two-way ANOVA followed by Bonferroni's test (significant at *P* < 0.05).

## 3. Results and Discussion

The vertebrate circadian system consists of an element that detects environmental light, an internal oscillator, and one or more output signals. The internal oscillator is comprised by a central clock and several peripheral clocks which, when in synchrony, display a time-accurate output [53]. Notably, the central clock remains rhythmic when in culture [54], whereas peripheral clocks lose this ability due to the loss of coupling among cells [55, 56].

**Figure 1:**
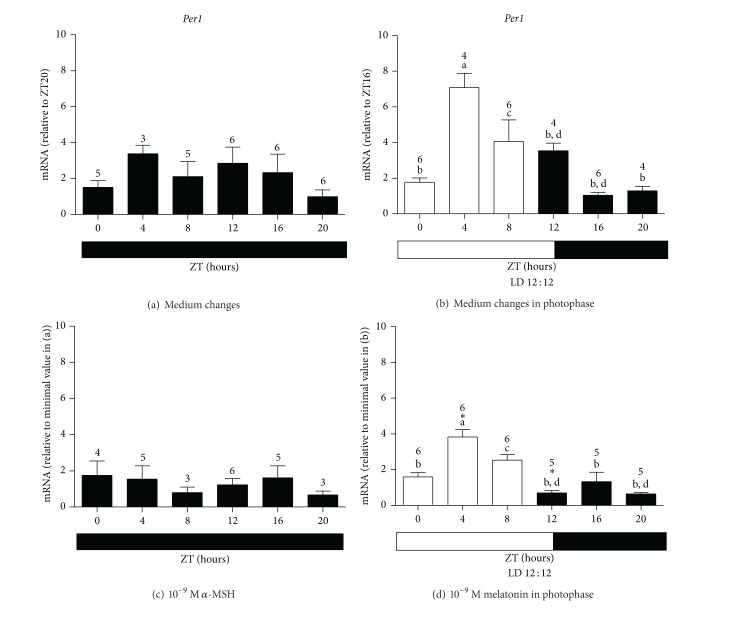
Quantitative PCR of* Per1* in* X. laevis* melanophores after 2 medium changes in DD (a); 2 medium changes in LD 12 : 12 (b); 10^−9^ M *α*-MSH in DD (c); and 10^−9^ M melatonin in the photophase of LD 12 : 12 (d). Each bar represents the mean, ± SE, mRNA relative to the noted ZT. (a) Significantly different from (b); (c) significantly different from (d) by one-way ANOVA, Tukey's post-test; ∗ means significantly different from the respective control at the same ZT, by two-way ANOVA and Bonferroni's post-test. *n* number shown on top of each bar in this and the following figures.

*Per1* expression in* X. laevis* melanophores kept in constant dark (DD) did not statistically vary throughout ZTs after medium changes (Figure 1(a)). Several data from literature have shown that a single medium change, serum shock [57, 58], phorbol esters (TPA) [59], or glucocorticoids [60] act as coupling factor in a variety of cultured cell types leading to a synchronization of clock genes. Unlike the literature reports, our data have shown that medium changes were unable to induce* Per1* temporal oscillation in* X. laevis* melanophores in DD, similarly to previous results in cells which were kept undisturbed in DD, that is, did not receive medium changes [45, 61]. However, when these cells were kept in light-dark cycles (12 : 12 LD) and subject to medium changes,* Per1* mRNA showed a temporal oscillation. Higher levels of mRNA were found at ZT4 comparing to all other ZTs (Figure 1(b), *P* < 0.001), and at ZT8 when was compared to ZT12 and ZT20 (Figure 1(b), *P* < 0.01).* Per1* mRNA has also been reported to increase in undisturbed* X. laevis* melanophores in LD 14 : 10 [61] or LD 12 : 12 [45] cycles. The results of the present study together with previous data suggest a major role for light in the clock gene expression changes in these cells, as it had already been demonstrated in* X. laevis* eye [62].

Because of the similar effects of light and of *α*-MSH on melanin granule dispersion, we evaluated the action of this hormone on one of the clock genes,* Per1*, as well as the effects of melatonin, an internal dark messenger for the organism. Melatonin was applied at the beginning of the photophase for 6 hours, whereas *α*-MSH was applied for 12 hours at the beginning of the subjective photophase in DD, thus mimicking the presence of light in causing melanin dispersion. One-way ANOVA analyses showed that there was no temporal variation in either 10^−9 ^M *α*-MSH treated cells or in cells subject to medium changes in DD. On the other hand, two-way ANOVA showed that the hormone treatment considerably affected the results (*F*_(1,45)_ = 10.19, *P* = 0.0026, Figures 1(a) and 1(c)), though no effects were seen when each time-point comparison was analyzed by Bonferroni's post-test.

**Figure 2:**
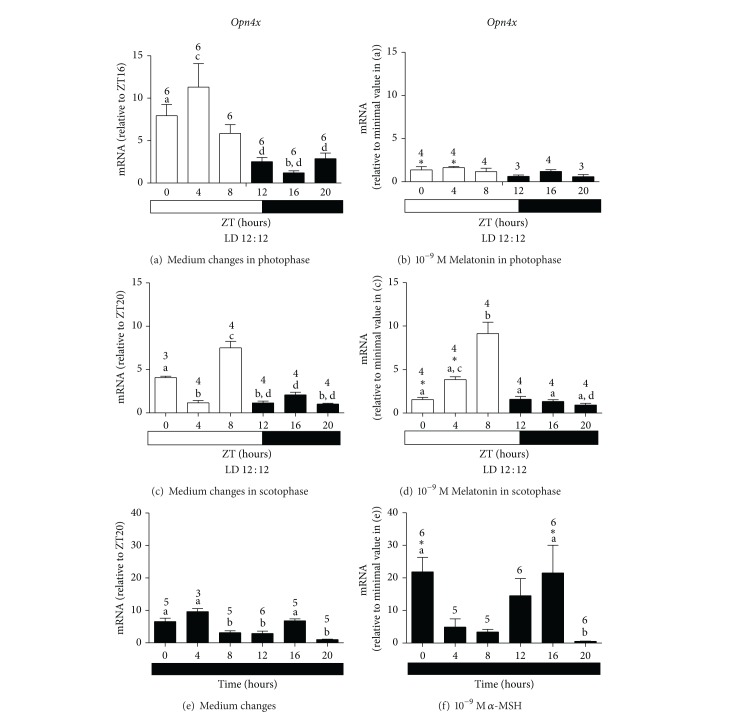
Quantitative PCR of* Opn4x *in* X. laevis* melanophores after 2 medium changes in the photophase of LD 12 : 12 (a); 10^−9^ M melatonin in the photophase of LD 12 : 12 (b); 2 medium changes in the scotophase of LD 12 : 12 (c); 10^−9^ M melatonin in the scotophase of LD 12 : 12 (d); 2 medium changes in DD (e); 10^−9 ^M *α*-MSH in DD (f). Each bar represents the mean, ± SE, mRNA relative to the noted ZT. (a) Significantly different from (b); (c) significantly different from (d), except in 2 C where (a) is different from (b) and (c) is different from (a) and (d) by one-way ANOVA and Tukey's post-test; ∗ means significantly different from the respective control at the same ZT, by two-way ANOVA and Bonferroni's post-test.

**Figure 3:**
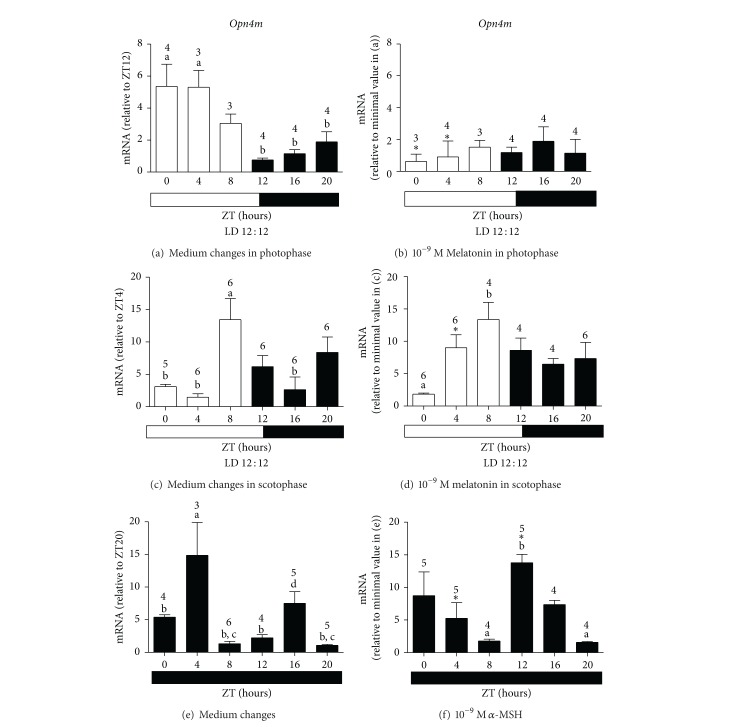
Quantitative PCR of* Opn4m *in* X. laevis* melanophores after to 2 medium changes in the photophase of LD 12 : 12 (a); 10^−9 ^M melatonin in the photophase of LD 12 : 12 (b); 2 medium changes in the scotophase of LD 12 : 12 (c); 10^−9 ^M melatonin in the scotophase of LD 12 : 12 (d); 2 medium changes in DD (e); 10^−9 ^M *α*-MSH in DD (f). Each bar represents the mean, ± SE, mRNA relative to the noted ZT. (a) Significantly different from (b); (c) significantly different from (d), by one-way ANOVAandTukey's post-test; ∗ means significantly different from the respective control at the same ZT, by two-way ANOVA and Bonferroni's post-test.

The temporal oscillations of* Per1* expression seen in control cells persisted after melatonin treatment. Higher levels of* Per1* mRNA were seen at ZT4 when compared to ZT0, ZT12, ZT16, and ZT20 (*P* < 0.001, Figure 1(d)), and at ZT8 as compared to ZT12 and ZT20 (*P* < 0.01, Figure 1(d)). Melatonin and time exerted strong influences on the results, as shown by two-way ANOVA (*F*_(1,52)_ = 20.06, *P* < 0.0001 and *F*_(5,52)_ = 19.74, *P* < 0.0001, resp.). Bonferroni's post-test analysis of each time point indicated that* Per1 *mRNA levels were 2 times lower at ZT4 (*P* < 0.001) and ZT12 (*P* < 0.01) after 10^−9^ M melatonin treatment in the photophase (Figure 1(d)) than the control group (Figure 1(b)). Interestingly, the temporal oscillation of* Per1* expression seen in the control was maintained. Bluhm and coworkers [61] found that 1 hour melatonin treatment of* X. laevis* melanophores, kept in DD, did not affect* Per1* expression, except for an increase at 3 hours. In mammals, entrainment mechanisms involve light stimulation of melanopsin positive ganglion cells that release glutamate/PACAP in the SCN cells, ultimately increasing* Per1* expression [63]. In undisturbed* X. laevis* melanophores, light at 460 nm wavelength, which maximally stimulates the melanopsins (or one of them) [42], also leads to enhancement of* Per1* expression [45]. These results point to a role of a blue light sensitive pigment in the light entrainment mechanism of* X. laevis *melanophores. Thus, we hypothesized that melatonin decreasing effects on the amplitude of* Per1* expression found here could be due to melatonin-induced reduction of the photopigment expression. In fact, 10^−9^ M melatonin treatment in the photophase dramatically decreased* Opn4x* and* Opn4m* expression during the light period, as compared to cells only subject to medium changes (Figures 2(a) and 2(b); Figures 3(a) and 3(b)) as described below.

Melatonin treatment in the photophase considerably affected* Opn4x* (*F*_(1,73)_ = 16.49, *P* < 0.0001 and *F*_(5,73)_ = 2.84, *P* = 0.0214 resp.) and* Opn4m* (*F*_(1,74)_ = 21.48, *P* < 0.0001 and *F*_(5,74)_ = 3.64, *P* = 0.0053 resp.) expressions. In addition, the temporal oscillation of* Opn4x* and* Opn4m* seen after medium changes in the photophase (Figures 2(a) and 3(a)) was abolished by the hormone treatment (Figures 2(b) and 3(b)). This reduction was statistically significant as shown by Bonferroni post-test for* Opn4x* and* Opn4m* at ZT0 (*P* < 0.05, *P* < 0.001 resp.) and ZT4 (*P* < 0.001). Therefore, melatonin is affecting the sensitivity of* X. laevis* melanophores to an important light-wavelength (460–480 nm), which we believe is the entrainment factor in this model. Melatonin* per se* does not seem to participate in the entrainment mechanism of the biological clock, as demonstrated by its lack of effect on* Per1* expression when applied either in the photophase (Figure 1(d)), or in the scotophase (data not shown).

If medium changes were performed in the photophase,* Opn4x* and* Opn4m,* showed a temporal profile with higher expression throughout the light period (Figures 2(a) and 3(a)).* Opn4x* expression was higher at ZT0 as compared to ZT16 (*P* < 0.05) and at ZT4 as compared to ZT12, ZT16, and ZT20 (*P* < 0.01).* Opn4m* expression was higher at ZT0 (*P* < 0.05) and at ZT4 (*P* < 0.01) than at ZT12, 16 and 20. When medium changes were performed in the scotophase, the highest levels of* Opn4x* and* Opn4m* mRNA were found at ZT8 (*P* < 0.01 and *P* < 0.05 resp.), that is, at the end of the light period (Figures 2(c) and 3(c)). The profile seen for* Opn4x* (Figure 2(a)) did not differ from what has been reported by Moraes and coworkers in* X. laevis* melanophores subject to 12 : 12 LD without further manipulation, whereas for* Opn4m,* LD cycles were not sufficient to induce temporal changes (unpublished data). So,* Opn4m* requires LD cycles plus medium changes to display a temporal oscillation as shown here (Figure 3(a)). Therefore, light seems to be the important* zeitgeber* for* Opn4x*, since it already showed an oscillatory pattern, which was not affected by medium changes, similarly to what was observed in* Per1 *expression.

Melatonin applied in the scotophase strongly affected* Opn4x* and* Opn4m* expressions throughout the time points (*F*_(5,35)_ = 60.7, *P* < 0.0001 and *F*_(5,32)_ = 10.96, *P* < 0.0001 resp.), which implied an altered temporal profile of both melanopsins.* Opn4x* mRNA levels decreased at ZT0 (*P* < 0.05) and increased at ZT4 (*P* < 0.001, Figure 2(d)), as compared to cells only subject to medium changes in the scotophase (Figure 2(c)).* Opn4m* mRNA levels increased at ZT4 (*P* < 0.05, Figure 3(d)), as compared to cells in control group (Figure 3(c)). Melatonin seems to exert permissive actions on gene expression, allowing* Opn4x* and* Opn4m* to resume a smoother time-course variation (Figures 2(d) and 3(d)). In control cells subject to medium changes in LD,* Opn4x* mRNA levels were higher at ZT8 compared to all other ZTs (*P* < 0.001), and* Opn4m* mRNA levels were higher at ZT8 compared to ZT0, ZT4, and ZT16 (*P* < 0.05). After melatonin treatment, both melanopsin expressions gradually increased during the photophase.

Melatonin administration during the scotophase mimics pineal melatonin secretion in physiological conditions, so one would expect a more relevant effect of melatonin. However, this hormone was incapable of significantly altering melanopsin temporal oscillations. Both diurnal and nocturnal animals release pineal gland melatonin at night, indicating the duration of the dark phase of the day, thus melatonin is a crucial compound that sets circadian temporal system [64]. During the photophase, melatonin seems to promote a disruption of the light sensor —melanopsins in this model— represented by the dramatic inhibition of both photopigment expressions and loss of changes in the temporal profile (Figures 2(a), 2(b), 3(a), and 3(b)). This inhibitory effect of melatonin on gene expression was also seen after only 1 hour melatonin treatment of* X. laevis* melanophores in DD [61].* X. laevis* melanophores, therefore, is an interesting model to study melatonin regulation of the circadian peripheral systems.

*α*-MSH exerts opposite effect on* Opn4x* expression when compared to melatonin, as it does on pigment translocation. After 10^−9 ^M *α*-MSH treatment,* Opn4x* expression increased 3-4 times at ZT0 (*P* < 0.05) and ZT16 (*P* < 0.05). The stimulatory effect of *α*-MSH on the overall expression was considered very significant (*F*_(1,51)_ = 7.7, *P* = 0.0077) (Figures 2(e) and 2(f)).* Opn4m*, on the other hand, was less affected by *α*-MSH; its mRNA levels decreased at ZT4 (*P* < 0.05) and increased at ZT12 (*P* < 0.001) as compared to the control group (Figures 3(e) and 3(f)). After *α*-MSH or medium changes, both* Opn4x* and* Opn4m* showed temporal oscillation. In DD after medium changes, similar levels of* Opn4x* mRNA were found at ZT0, ZT4, and ZT16, compared to ZT8, ZT12, and ZT20 (*P* < 0.05) (Figure 2(e)); but higher levels were seen at ZT0 and ZT16 compared to ZT20 after *α*-MSH treatment (*P* < 0.05, Figure 2(f)). In DD after medium changes,* Opn4m* expression was higher at ZT4 as compared to ZT0, ZT8, ZT12, and ZT20 (*P* < 0.05) and lower at ZT8 as compared to ZT16 (Figure 3(e)); after *α*-MSH treatment, its expression was higher at ZT12 as compared to ZT8 and ZT20 (*P* < 0.01) (Figure 3(f)). Therefore *α*-MSH effects on melanopsins expressions do not seem to involve the molecular clock machinery, since the hormone did not affect* Per1 *expression except for a sporadic modulation throughout the day.

In fact, the opposite effects of *α*-MSH and melatonin seem to be conserved, because they have previously been described in other groups of animals [65, 66]. This may be explained by the opposite actions of these hormones on cAMP production in* X. laevis* melanophores: *α*-MSH increases the nucleotide concentration [43], whereas melatonin reverses melanosome dispersion through the inhibition of adenylyl cyclase [67].

We would expect similar effects of *α*-MSH (Figures 2(f) and 3(f)) and light (Figures 2(a) and 3(a)) on* Opn4x* and* Opn4m* expression; however, unlike the hormone, light was able to induce temporal variation in both melanopsin expressions. This may be explained by the fact that the signaling pathway leading to light-stimulated responses of gene expression and pigment dispersion is distinct from the one triggering melanin dispersion by *α*-MSH. In fact, light stimulates PKC pathway leading to an increase of cytosolic Ca^2+^ and cGMP [42], whereas *α*-MSH promotes an increase of cAMP [43]. This response is decreased by preexposure of melanophores to light, because light-induced Ca^2+^ rise inhibits adenylyl cyclase [43]. Although mammalian* Per1* promoter is known to possess cAMP responsive element [68], little is known about* X. laevis Per1* promoter. In* Danio rerio* embryonic cells, we and others have demonstrated that the light-stimulated pathway increases both* Per1* and* Per2 *expressions, what is blocked by MAP kinase inhibitors [69–71], whereas there is controversial evidence of cAMP involvement [71]. In NIH-3T3 fibroblasts, it has been shown that the circadian oscillation of clock gene expression is abolished by a MEK inhibitor [59], suggesting that transcription factors other than CREB may be the activators of* Per1* promoter in vertebrate peripheral clocks.

In summary,* Per1* is sensitive to light-dark cycles, regardless of the presence of melatonin or *α*-MSH, which slightly inhibited its expression. It is worth to mention, however, that the hormones may be affecting the expression of other clock genes, such as* Per2* or* Bmal1*, or even exerting a posttranslational effect. Melatonin effects on melanopsins depend on the time of application. Melatonin applied in the photophase dramatically decreases* Opn4x* and* Opn4m* expressions and abolishes its temporal oscillation, opposite to *α*-MSH which increases the melanopsin expressions. Our results demonstrate that unlike what has been reported for other peripheral clocks and cultured cells, medium changes or hormones do not play a major role in synchronizing* Xenopus* melanophore cell population. This difference is most probably due to the fact that* X. laevis* melanophores possess functional photopigments, the melanopsins, enabling these cells to respond primarily to light, which triggers melanin dispersion and modulation of gene expression.

## Figures and Tables

**Table 1:** Primers and probes used for qPCR assays.

*18S RNA *	Forward: 5′-CGGCTACCACATCCAAGGAA-3′
	Backward: 5′-GCTGGAATTACCGCGGCT-3′
	Probe: 5′-5-TexRd-TGCTGGCACCAGACTTGCCCTC-BHQ_2-3′

*Opn4x * NM_00108564.1	Forward: 5′-ATTATTGTCCTTGTCTGGATGTATTCA-3′
	Backward: 5′-AAGCCCTCTGGCACATAGGAA-3′
	Probe: 5′-6-FAM-AATGTGGAGCTTGGCACCATTACTTGGC-BHQ_1-3′

*Opn4m* DQ_384639.1	Forward: 5′-AGGGCAGTGCTAATCCTTTCAGGT-3′
	Backward: 5′-AATCCCAGGTGCAGGATGTCAGAA-3′

*Per1* NM_001085703.2	Forward: 5′-TGAAGGCCCTTAAAGAGCTAAAGA-3′
	Backward: 5′-TTGCCAGTGTGCCAGACTTG-3′
	Probe: 5′-/5Cy5/TCGGCTCCCATCAGAGAAGAG GCTAAAAG/3BHQ_2/3′
